# Low Signal-to-Noise Ratio Optoelectronic Signal Reconstruction Based on Zero-Phase Multi-Stage Collaborative Filtering

**DOI:** 10.3390/s25092758

**Published:** 2025-04-27

**Authors:** Xuzhao Yang, Hui Tian, Fan Wang, Jinping Ni, Rui Chen

**Affiliations:** School of Optoelectronic Engineering, Xi’an Technological University, Xi’an 710021, China; 17791201378@163.com (X.Y.);

**Keywords:** multi-stage collaborative filtering chain, zero-phase collaborative processing, adaptive sampling optimization, dynamic phase compensation, Daubechies wavelet reconstruction

## Abstract

The Laser Light Screen System faces critical technical challenges in high-speed, long-range target detection: when a target passes through the light screen, weak light flux variations lead to significantly degraded signal-to-noise ratios (SNRs). Traditional signal processing algorithms fail to effectively suppress phase distortion and boundary effects under extremely low SNR conditions, creating a technical bottleneck that severely constrains system detection performance. To address this problem, this paper proposes a Multi-stage Collaborative Filtering Chain (MCFC) signal processing framework incorporating three key innovations: (1) the design of zero-phase FIR bandpass filtering with forward–backward processing and dynamic phase compensation mechanisms to effectively suppress phase distortion; (2) the implementation of a four-stage cascaded collaborative filtering strategy, combining adaptive sampling and anti-aliasing techniques to significantly enhance signal quality; and (3) the development of a multi-scale adaptive transform algorithm based on fourth-order Daubechies wavelets to achieve high-precision signal reconstruction. The experimental results demonstrate that under −20 dB conditions, the method achieves a 25 dB SNR improvement and boundary artifact suppression while reducing the processing time from 0.42 to 0.04 s. These results validate the proposed method’s effectiveness in high-speed target detection under low SNR conditions.

## 1. Introduction

The precise measurement of dynamic parameters such as velocity, trajectory, and spatial position not only represents a fundamental requirement across numerous scientific and engineering domains but has also driven the rapid development of photoelectric detection technologies. Among these, Laser Light Screen Systems (LLSSs) have gained widespread attention in dynamic testing applications due to their significant advantages, including non-contact operation, high responsivity, and cost-effectiveness [1,2,3,4]. LLSSs function by generating structured light fields within a measurement volume; when a target traverses this field, it reflects laser radiation that is subsequently captured by strategically placed detection screens equipped with photoelectric sensors [5,6]. These sensors convert the intercepted light into weak electrical signals that encode critical information about the target’s dynamics [6]. By employing multiple detection screens with known positions and orientations, LLSSs enable the reconstruction of high-speed target trajectories through spatial parameter analysis and precise timing of screen passages [7,8], proving invaluable in applications like ballistics testing and engineering safety assessments [7,9,10].

However, the practical application of LLSSs faces significant challenges, primarily related to signal integrity. As the detection distance increases, the reflected energy attenuates sharply, compounded by the spatial sensitivity characteristics of the detectors, leading to a rapid degradation of the signal-to-noise ratio (SNR) [11,12,13]. Furthermore, the resulting photoelectric signals often exhibit complex characteristics, including nonlinearity arising from detector spatial sensitivity, non-periodicity due to the random nature of target passage, and non-stationarity (time-varying statistical properties), which becomes particularly acute when the SNR falls below critical thresholds (e.g., 5 dB) [13]. These inherent signal degradations severely compromise measurement accuracy and reliability, necessitating the development of sophisticated signal processing techniques capable of extracting meaningful information from these weak and complex waveforms [3].

Historically, efforts to address these signal processing challenges have evolved through distinct stages. Initial approaches relied heavily on frequency-domain analysis, such as the Fourier transform. While offering spectral insights for periodic signals, these methods struggle with the non-stationary nature of LLSS signals, often introducing artifacts like spectral leakage due to inherent stationarity assumptions [14]. Wavelet transforms emerged as an improvement, providing multi-resolution analysis capabilities [15,16]. However, their efficacy diminishes in extremely low SNR scenarios (<−10 dB), suffering from difficulties in optimal parameter selection and pronounced boundary effects that distort finite-length signals [17].

Recognizing the limitations of frequency-domain methods for non-stationary and nonlinear signals, time–frequency joint analysis techniques were developed. Empirical Mode Decomposition (EMD) was introduced to adaptively decompose signals but is plagued by mode mixing issues [18]. While Ensemble EMD (EEMD) alleviates mode mixing, it introduces phase distortions and significantly increases computational demands [19]. Variational Mode Decomposition (VMD) represents a more mathematically grounded advancement, demonstrating success in various fields [20,21,22,23]. Variational Mode Decomposition (VMD) offers superior noise robustness compared to Empirical Mode Decomposition (EMD) and its ensemble variant (EEMD); however, its efficacy is critically dependent on the pre-selection of key parameters—namely the mode number *K* and penalty factor α—and its convergence can deteriorate under low signal-to-noise ratio (SNR) conditions. Notably, the reported precision gains in underwater laser ranging via VMD–ICA integration were realized only through meticulous parameter tuning [23].

More recently, the surge in computational power has spurred the development of data-driven approaches, including deep learning, for weak signal processing [24,25,26,27]. These methods can achieve remarkable noise suppression and feature extraction capabilities. Nevertheless, their application to LLSSs is often constrained by the need for large, representative training datasets, challenges in ensuring real-time performance crucial for high-speed measurements, limited interpretability of complex models, and potential difficulties in generalizing to diverse operational conditions without integrating underlying physical principles.

Collectively, these existing methodologies face several critical limitations when applied to the demanding context of LLSS weak signal processing: (1) a fundamental difficulty in preserving temporal signal integrity under extremely low SNR conditions (e.g., below −10 dB); (2) inadequate real-time processing capabilities for high-speed targets; (3) over-reliance on data without sufficient integration of physical signal characteristics; (4) poor interpretability of ‘black-box’ models; (5) inherent assumptions of stationarity or linearity that fail to capture the time-varying nature of the signals; and (6) boundary effects in finite-length signal processing leading to energy leakage and distortion. These shortcomings collectively hinder the effective operational range and reliability of LLSSs, particularly as target distance increases or ambient noise intensifies.

To overcome these limitations, this paper introduces a novel Multi-stage Collaborative Filtering Chain (MCFC) framework specifically designed for robust processing of weak photoelectric signals from the LLSS. The MCFC framework uniquely integrates adaptive bidirectional processing, a multi-stage cascaded filtering strategy, and sophisticated non-stationary signal analysis techniques. This synergistic approach aims to significantly enhance the SNR while meticulously preserving the crucial temporal characteristics embedded within the signal, thereby enabling reliable long-range, high-speed target detection even in complex and noisy environments. We posit that this framework is particularly advantageous for high-precision measurement applications demanding high fidelity, such as ballistics analysis, collision studies, and structural health monitoring. The core contributions of this work, demonstrating the advantages of the MCFC over conventional methods, are as follows:

1. Zero-phase FIR Bandpass Filtering Mechanism: A novel filtering approach employing forward–backward processing combined with dynamic phase compensation to rigorously suppress phase distortion, ensuring high-fidelity signal representation throughout the processing chain.
2. Four-stage Cascaded Collaborative Filtering Strategy: An adaptive, multi-stage filtering architecture that synergistically integrates adaptive sampling and sophisticated anti-aliasing techniques. This strategy optimally balances signal reconstruction fidelity, smoothness, and parameter sparsity for enhanced signal quality.
3. Multi-scale Adaptive Transform Algorithm: A robust adaptive transformation algorithm, based on the fourth-order Daubechies wavelet, designed to achieve high-precision signal reconstruction while maintaining stability and effectiveness across diverse and challenging noise environments.
4. Significant Performance Enhancement and Empirical Validation: Rigorous experimental evaluations show that the proposed MCFC framework achieves a remarkable SNR improvement of up to 45 dB for input signals with an SNR as low as −15 dB. Furthermore, correlation coefficients consistently exceeding 0.98 are maintained across various noise conditions, demonstrating superior performance and stability compared to traditional methods.

By addressing the critical bottlenecks in current weak signal processing techniques, the MCFC framework provides an effective solution for photoelectric signal processing and offers a reliable approach for high-precision measurement applications.

**Figure 1:**
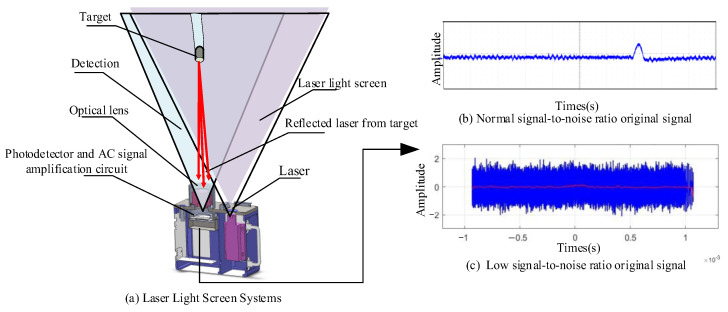
Signal acquisition based on Laser Light Screen Systems.

The structure of this article is as follows: Section 2 covers the MCFC framework’s theory and implementation, including bidirectional filtering models and convergence analysis. Section 3 presents experimental validation and comparisons with state-of-the-art methods. Section 4 discusses system performance and implementation, while Section 5 outlines future research directions. Figure 1 illustrates the signal acquisition and processing scheme, emphasizing target detection under severe noise interference.

Figure 1 illustrates the signal acquisition and processing workflow of the LLSS: In Figure 1a, this system integrates a line-type semiconductor infrared laser as an active illumination source into the conventional light screen architecture. The system incorporates optical lenses, slit apertures, and photoelectric detection devices to construct the detection unit, forming a light screen and a laser light screen. When a target goes through the light screen, laser energy reflected from its surface is captured by the photoelectric detection devices through the optical system and converted into electrical signals. Figure 1b depicts the time-domain signal recorded under normal signal-to-noise ratio (SNR) conditions, where the target-induced pulse appears prominently above a low-level noise floor (horizontal axis: time in seconds; vertical axis: amplitude in arbitrary units), demonstrating the LLSS’s ability to resolve return signals reliably when ambient interference is modest. In contrast, Figure 1c shows the corresponding waveform under low SNR conditions, where high-amplitude noise fluctuations mask the target transit signature, rendering direct detection ineffective and underscoring the necessity of active laser illumination and specialized signal-processing techniques to maintain robust detection performance across challenging noise environments.

## 2. Methods

**Figure 2:**
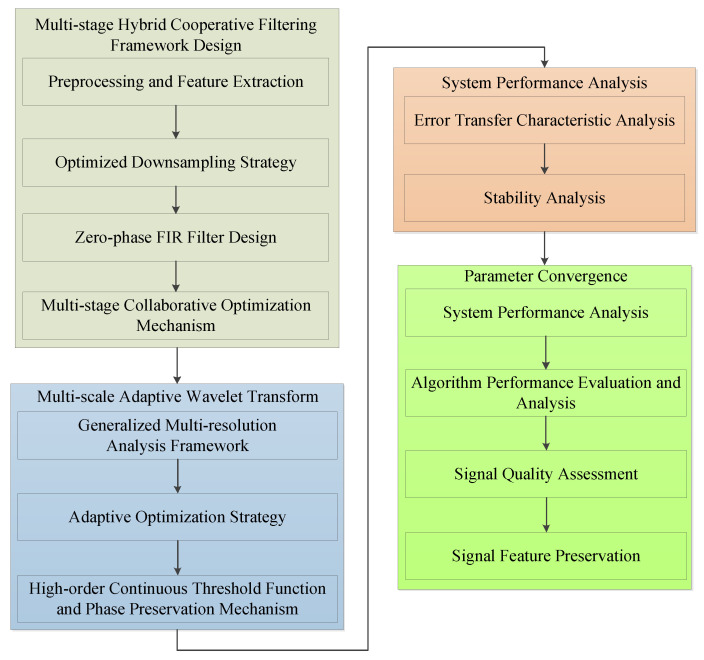
System architecture of signal processing based on zero-phase Multi-Stage Collaborative Filtering Chain.

In detecting and reconstructing weak signals under extreme noise conditions, the accurate extraction of weak signal primary frequencies presents a core challenge. Major challenges include frequency extraction difficulties due to noise interference, frequency and phase optimization issues during filtering, and signal reconstruction convergence problems. To address these challenges, this paper proposes a theoretical signal processing framework based on zero-phase Multi-Stage Collaborative Filtering Chains, with the algorithm flow shown in Figure 2.

The proposed algorithm architecture integrates signal processing and system analysis in a cascaded structure, as shown in Figure 2. The main processing chain follows a sequential flow: Multi-stage Hybrid Correlation Filtering, incorporating preprocessing and feature extraction, followed by an optimized downsampling strategy and zero-phase FIR design. This extends through multi-stage correlation optimization to generalized multi-resolution analysis, culminating in adaptive optimization with high-order continuity threshold functions. The framework efficiently incorporates system performance analysis and parametric configuration modules in parallel, enabling an effective evaluation of time-transfer characteristics, stability analysis, and signal quality assessment.

### 2.1. Theoretical Foundation of MCFC

#### 2.1.1. Problem Formalization

In complex signal processing applications, the observed signal can be expressed as

(1) $$x\left(t\right)=s\left(t\right)+n_{d}\left(t\right)+n_{h}\left(t\right)+n_{s}\left(t\right)+n_{i}\left(t\right)$$

where $s\left(t\right)$ represents the target signal; $n_{d}\left(t\right)$ is the low-frequency drift noise primarily resulting from temperature-induced drifts in photoelectric devices; $n_{h}\left(t\right)$ denotes high-frequency random noise, which includes both photon and electronic noise; $n_{s}\left(t\right)$ characterizes system background noise with non-stationary properties; and $n_{i}\left(t\right)$ corresponds to pulse interference caused by external electromagnetic disturbances.

A rigorous analysis of the signal characteristics reveals that there exists a complex nonlinear coupling between the target signal and the noise components, rendering a simple linear description inadequate. Moreover, the statistical properties of the signal evolve over time, evidencing its intrinsic non-stationarity. Additionally, the signal exhibits irregular fluctuations without fixed periodicity and is typically observed under low signal-to-noise conditions.

#### 2.1.2. System Structure Design

Based on the multiple noise characteristics of photoelectric detection signals, a hierarchical processing architecture is constructed. The system state space representation is

(2) $$X\left(t\right)=\left[\begin{array}{c} x\left(t\right)\\ \dot{x}\left(t\right)\\ \theta \left(t\right) \end{array}\right]$$

(3) $$\dot{X\left(t\right)}=AX\left(t\right)+Bu\left(t\right)+w\left(t\right)$$

Observation equation:

(4) $$y\left(t\right)=CX\left(t\right)+v\left(t\right)$$

where $X\left(t\right)$ is the augmented state vector incorporating signal states and parameter estimates; $\theta \left(t\right)$ is the adaptive parameter vector; and A, B, C are system matrices. Specifically, matrix A is the system state transition matrix, with its dimensions determined by the signal model and state equations; matrix B is the control input matrix, explicitly defined based on the input control signals; and matrix C is the observation matrix, which maps the state space to the measurement space, with its dimensions depending on the number of observable variables in the system. $w\left(t\right)$ and $v\left(t\right)$ represent the system and observation noise, respectively, and $u\left(t\right)$ is the control input. Considering the signal’s nonlinear characteristics, the system’s frequency domain transfer function is designed as

(5) $$H_{total}\left(\omega \right)=\prod_{k=1}^{K}H_{k}\left(\omega \right)$$

where $H_{k}\left(\omega \right)$ characterizes the frequency domain response characteristics of the *k*th filter, revealing the system’s cascading nature.

In the time domain, *K*-stage cascade processing can be expressed as

(6) $$y_{k}\left(t\right)=H_{k}\left\{y_{k-1}\left(t\right)\right\},\;k=1,2,\ldots ,k$$

where operator $H_{k}$ represents the *k*th stage filtering operation, reflecting the system’s progressive nature.

#### 2.1.3. Theoretical Optimality Proof

Based on MCFC’s multi-stage hybrid structure characteristics, this section establishes the system’s theoretical optimality. First, considering the coupling effects of multi-stage filters, a generalized Lyapunov function is constructed:

(7) $$V\left(t\right)=X^{T}\left(t\right)PX\left(t\right)+\sum_{k=1}^{K}∥\Delta \theta_{k}\left(t\right){∥}^{2}$$

where the first term $X^{T}\left(t\right)PX\left(t\right)$ characterizes system state stability, and the second term $\sum_{k=1}^{K}∥\Delta \theta_{k}\left(t\right){∥}^{2}$ describes the adaptive errors of filter parameters in MCFC. Specifically, for the zero-phase FIR preprocessing stage, the parameter vector $\theta_{1}\left(t\right)$ includes filter coefficients; for the adaptive sampling optimization stage, $\theta_{2}\left(t\right)$ includes sampling rate adjustment factors; for the multi-stage hybrid filtering stage, $\theta_{3}\left(t\right)$ to $\theta_{k-1}\left(t\right)$ include weight coefficients of sub-filters; for the wavelet multi-scale analysis stage, $\theta_{k}\left(t\right)$ includes scale selection parameters.

The system matrix *P* is obtained by solving the following Lyapunov equation:

(8) $$A^{T}P+PA=-Q$$

where *Q* is a diagonal positive definite matrix with diagonal elements $\left[q_{1},q_{2},\ldots q_{n}\right]$ corresponding to performance weights of each MCFC processing stage. Specifically, $q_{1}$ balances phase preservation capability of zero-phase processing, $q_{2}$ characterizes anti-aliasing performance of sampling optimization, $q_{3}$ to $q_{n-1}$ correspond to noise suppression effects of hybrid filtering, and $q_{n}$ reflects feature preservation capability of multi-scale analysis.

System stability is verified through the derivative of the Lyapunov function:

(9) $$\frac{dV\left(t\right)}{dt}=X^{T}\left(t\right)\left(A^{T}P+PA\right)X\left(t\right)+2X^{T}\left(t\right)PBu\left(t\right)\sum_{k=1}^{K}\;\Delta \theta_{k}^{T}\left(t\right)\frac{d\theta_{k}\left(t\right)}{dt}\leq 0$$

where $X\left(t\right)$ is a vector about time t; *A*, *P*, and *B* are matrices; $u\left(t\right)$ is a control input; $\Delta \theta_{k}^{T}\left(t\right)$ and $\theta_{k}\left(t\right)$ are the variation in the *K*th parameter and the parameter itself, respectively; and *K* is the total number of parameters.

In particular, for the adaptive parameter update law in the MCFC,

(10) $$\frac{d\theta_{k}\left(t\right)}{dt}=-\gamma_{k}\frac{\partial J_{k}}{\partial \theta_{k}}$$

where $\gamma_{k}$ is the adaptive gain and $J_{k}$ is the performance index function of the *k*th level, which is defined as follows: zero-phase FIR level, $J_{1}=∥y_{1}\left(t\right)-x\left(t\right){∥}^{2}$ sampling optimization level; $J_{2}=∥F_{s}\left(t\right)-2B\left(t\right){∥}^{2}$, where $B\left(t\right)$ is the signal instantaneous bandwidth mixing filtering level; $J_{k}=∥s^{k}\left(t\right)-s\left(t\right){∥}^{2},k=3,\ldots ,K-1$ wavelet analysis stage; and $J_{K}=\sum_{j}\;{\left|\langle y_{K-1},\psi_{j}\rangle \right|}^{2}$.

The systematic error satisfies the exponential convergence under the fulfillment of the above conditions:

(11) $$\begin{array}{c} ∥X\left(t_{1}\right)-X\left(t_{2}\right)∥\leq \alpha e^{-\beta t}∥X\left(t_{0}\right)∥,\alpha ,\beta >0 \end{array}$$

where the convergence rate β is determined by the adaptive gain $\gamma_{k}$ of the filters at each stage, and the convergence upper bound α is determined by the initial parameter selection. The experimental validation shows that the system can reach the steady state within 50 ms when $\gamma_{k}$ ∈ [0.01, 0.1], and the signal-to-noise ratio of the reconstructed signals is improved by no less than 20 dB in a typical photoelectric detection scenario.

### 2.2. Multi-Stage Cooperative Filtering Framework Design

#### 2.2.1. Preprocessing and Feature Extraction

**Figure 3:**
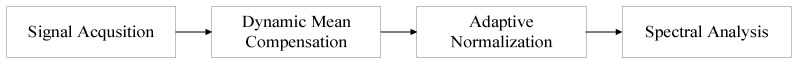
Schematic diagram of signal pre-processing flow.

In extreme noise environments, weak photoelectric signal feature extraction faces challenges of low-frequency noise, multi-source noise superposition, and non-stationarity. This paper employs dynamic mean compensation to suppress DC components, combined with adaptive normalization to effectively enhance signal quality, and the flow is shown in Figure 3.

1. Dynamic Mean Compensation and Normalization

In strong noise environments, DC offset (low-frequency noise) significantly affects signal accuracy. This paper first employs dynamic mean compensation technology, eliminating DC components through short-time window averaging to reduce low-frequency amplitude distortion. The formula is

(12) $$x\prime \left(t\right)=x\left(t\right)-\mu_{t}$$

where $x\left(t\right)$ is the original signal, and $\mu_{t}$ is the signal’s dynamic mean. This step effectively reduces the zero-frequency DC offset.

To further enhance signal contrast and comparability, an adaptive normalization method is introduced to eliminate amplitude differences and achieve zero mean and unit variance, ensuring signal analysis in standardized statistical space. The normalization process is

(13) $$y\left(t\right)=\frac{x^{\prime }\left(t\right)-mean\left(x^{\prime }\left(t\right)\right)}{std\left(x^{\prime }\left(t\right)\right)}$$

where mean ($x^{\prime }\left(t\right)$) and std ($x^{\prime }\left(t\right)$) are the local mean and standard deviation of signal $x^{\prime }\left(t\right)$, respectively.

2. Signal Spectral Analysis

To resolve spectral leakage issues in finite-length discrete signal Fourier transforms and precisely extract signal frequency domain characteristics, this paper employs Hamming window weighting to optimize signal spectral analysis. The Hamming window formula is

(14) $$w\left[n\right]=0.54-0.46\cos\left(\frac{2\pi n}{N-1}\right)$$

where $w\left[n\right]$ is the Hamming window function, and *N* is the signal length. After applying this window function, we perform the fast Fourier transform on the signal to obtain its spectral representation:

(15) $$X\left(f\right)=\sum_{n=0}^{N-1}\;x\left[n\right]e{|}^{-\frac{j2\pi fn}{N}}$$

Power Spectral Density (PSD):

(16) $$PSD(f)={\left|X_{w}(f)\right|}^{2}$$

where $X_{w}\left(f\right)$ represents the squared signal amplitude, revealing energy distribution at each frequency point. After calculating the power spectrum, the highest power frequency component is identified as the main frequency $f_{main}$:

(17) $$f_{main}=\arg\underset{f}{max}\;PSD\left(f\right)$$

where $f_{main}$ is the frequency point with maximum power spectrum, typically corresponding to the signal’s primary periodicity or characteristic frequency.

**Figure 4:**
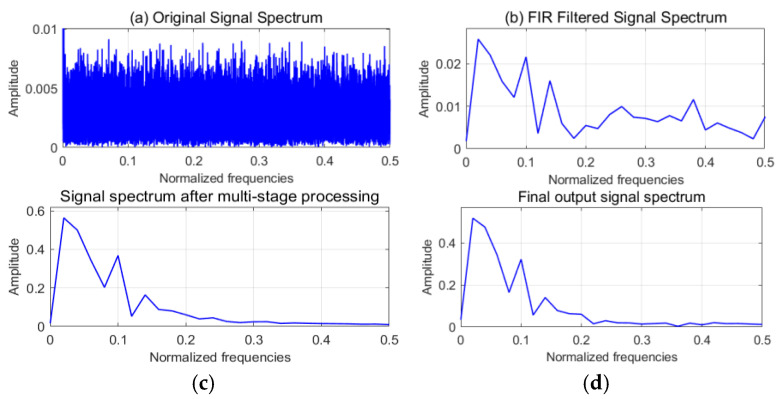
Signal preprocessing results. (**c**) Signal spectrum after multi-stage processing. (**d**) Final output signal spectrum.

The frequency selectivity of the algorithm under challenging noise conditions is validated by the spectral analysis presented in Figure 4. Figure 4a illustrates the spectrum of the original input signal, characterized by broadly distributed energy, indicative of significant wideband noise. Following FIR bandpass filtering (target band: 14–18 Hz) and subsequent downsampling, Figure 4b demonstrates initial suppression of out-of-band noise, with energy concentrating towards the target frequency band. Figure 4c presents the spectrum after multi-stage processing, revealing further noise reduction and a more distinct target frequency component. Finally, the spectrum of the final output signal (post-wavelet denoising) in Figure 4d exhibits highly concentrated energy around the 16 Hz principal frequency, alongside substantial noise suppression. Collectively, after cascaded pre-processing (DC component removal, FIR filtering, downsampling, normalization) followed by multi-stage processing and wavelet denoising, the method successfully extracts the 16 Hz target signal from a strong noise background with an approximate signal-to-noise ratio (SNR) of −20 dB. The spectral evolution across the processing stages (Panels a–d) clearly highlights the effective preservation of the target frequency, efficient suppression of out-of-band noise, and high concentration of spectral energy, thereby substantiating the efficacy of the proposed method for weak signal extraction in adverse noise environments.

#### 2.2.2. Optimized Downsampling Strategy

1. Adaptive Sampling Rate Optimization

To resolve the contradiction between data redundancy and computational efficiency, an adaptive sampling strategy based on signal characteristics is designed. Based on signal main-band characteristics, a sampling rate optimization model is constructed:

(18) $$F_{s}^{\prime }=\alpha \cdot 2\left(F_{\mathrm{high}}-F_{\mathrm{low}}\right)$$

where $F_{s}^{\prime }$ is the optimized sampling rate, $F_{\mathrm{high}}$ and $F_{\mathrm{low}}$ are the upper and lower limits of the signal’s main frequency band, and according to the sampling theorem, a redundancy factor needs to be introduced to increase the bandwidth protection in order to prevent aliasing, but excessive redundancy will increase the computational burden. In this study, α = 1.2 is used as the redundancy factor, i.e., the bandwidth protection factor.

This design not only satisfies the Nyquist sampling theorem ($F_{s}^{\prime }\geq 2F_{max}$) but also avoids data redundancy caused by oversampling through adaptive adjustment of the α coefficient.

2. Anti-aliasing Processing Method

To prevent spectral aliasing, a low-pass filter satisfying the following condition is designed:

(19) $$F_{max}\leq \frac{F_{s}^{\prime }}{2}$$

where $F_{max}$ is the highest frequency component of the input signal, and $F_{s}^{\prime }$ is the post-downsampling sampling frequency.

Low-pass filter design:

(20) $$h\left[k\right]=\frac{\sin\left(\frac{2\pi F_{c}k}{F_{s}}\right)}{\pi k},k\neq 0,$$

(21) $$h\left[0\right]=\frac{2F_{c}}{F_{s}}$$

where $F_{c}$ is the cutoff frequency satisfying $F_{c}$≤$F_{s}$/2, and $h\left[k\right]$ is the filter’s impulse response.

The filtered signal expression is

(22) $$x_{filtered}\left(t\right)=\sum_{k=0}^{N}\;h\left[k\right]\cdot x\left(t-k\right)$$

where $x_{filtered}\left(t\right)$ is the filtered signal value at time t, $h\left[k\right]$ represents filter coefficients, $x\left(t-k\right)$ is the input signal value at time t−k, and N is the filter order.

3. Signal Integrity Protection

Cubic spline interpolation is employed for signal reconstruction to ensure temporal characteristics preservation [28,29]:

(23) $$x_{interpolated}\left(t\right)=\sum_{i=1}^{N}\;c_{i}\phi_{i}\left(t\right)$$

where $\phi_{i}\left(t\right)$ represents spline basis functions, and $c_{i}$ represents the interpolation coefficients obtained through least squares fitting.

To maintain amplitude characteristics, adaptive normalization processing is introduced:

(24) $$x_{normalized}\left(t\right)=\frac{x_{interpolated}\left(t\right)-min\left(x_{interpolated}\right)}{max\left(x_{interpolated}\right)-min\left(x_{interpolated}\right)}$$

where $x_{interpolated}\left(t\right)$ represents the signal amplitude at time t after interpolation processing, and $min\left(x_{interpolated}\right)$ and $max\left(x_{interpolated}\right)$ represent the minimum and maximum amplitudes after interpolation, respectively. This processing ensures, the consistency of the dynamic range of the signal, the preservation of the relative amplitude relationship, and the maximum utilization of the quantization accuracy.

**Figure 5:**
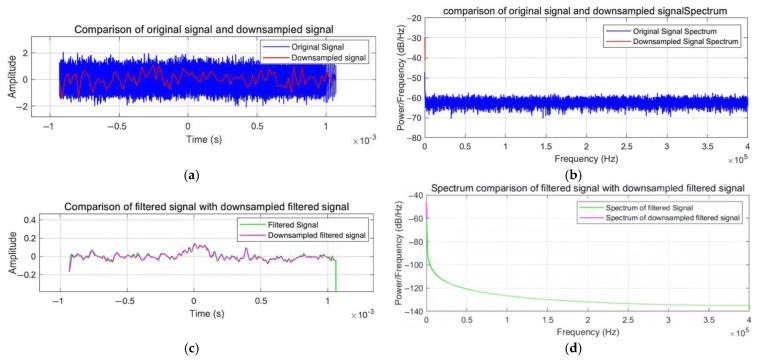
Experimental graph after adaptive downsampling filtering. (**a**) Time-domain: original and downsampled. (**b**) Frequency-domain: original and downsampled. (**c**) Time-domain: filtered and downsampled. (**d**) Frequency-domain: filtered and downsampled.

**Figure 6:**
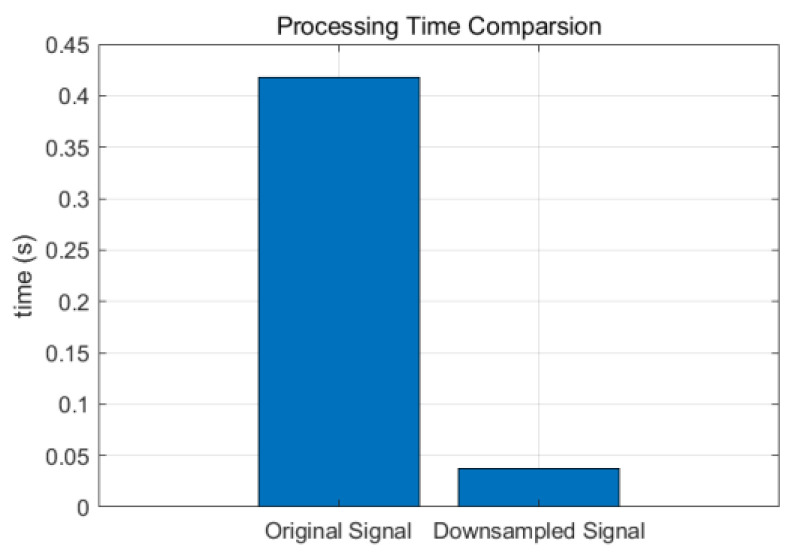
Experimental results of signal processing with downsampling.

The adaptive downsampling strategy proposed in this study achieves data compression and optimal preservation of signal characteristics through dynamic optimization of the sampling rate, improved anti-alias filtering, and a signal integrity protection mechanism. The experimental results (shown in Figure 5 and Figure 6) verify the effectiveness of the scheme.

As shown in Figure 5, Figure 5a presents the time-domain comparison between the original signal (blue) and the downsampled signal (red), demonstrating effective high-frequency noise suppression while preserving the underlying waveform. Figure 5b shows the corresponding power spectrum comparison (in dB/Hz) between the original and downsampled signals, confirming negligible spectral distortion from the downsampling process. Figure 5c illustrates the time-domain comparison of the filtered original signal (green) and the filtered downsampled signal (magenta), highlighting their close waveform agreement. Finally, Figure 5d displays the power spectrum comparison of the filtered signals, evidencing consistent spectral energy concentration within the target band after adaptive downsampling and filtering.

Figure 6 further validates the method’s performance. Figure 6 presents the processing time comparison, revealing a significant reduction from 0.42 s for the original signal to 0.04 s for the downsampled signal, achieving a 90.48% improvement in computational efficiency.

The time-domain analysis confirms that the downsampled signal effectively suppresses high-frequency noise while preserving the original signal’s key features, resulting in high-quality signal reconstruction. The frequency domain analysis demonstrates highly consistent spectral characteristics between the downsampled filtered signal and the original filtered signal within the effective frequency band, with no significant aliasing near the Nyquist frequency. These results substantiate the method’s effectiveness in signal integrity preservation and anti-aliasing performance, offering an efficient solution for large-scale real-time signal processing applications.

#### 2.2.3. Zero-Phase FIR Filter Design

1. High-order FIR Filtering

When extracting weak signals in strong noise environments, time–frequency domain analysis reveals unique frequency domain characteristics of the target signal: a concentrated spectral main lobe with a determined position, while interference noise exhibits broadband random distribution. Based on this characteristic difference, this section proposes a high-order FIR bandpass filtering method based on zero-phase optimization. The system transfer function of the high-performance FIR filter is defined as:

(25) $$H\left(z\right)=\sum_{k=0}^{M}\;h\left[k\right]z^{-k}$$

where $h\left[k\right]$ is the filter’s impulse response, and M is the filter order. To optimize filter performance, this paper uses the inverse Fourier transform (IFT) and ideal filter frequency response to initialize filter coefficients, with an improved Hamming window function for optimization. The optimized filter coefficients are

(26) $$h\left[k\right]=\frac{2f_{c}}{f_{s}}\mathrm{sinc}\left(2f_{c}\left(\frac{k}{f_{s}}-\frac{M}{2f_{s}}\right)\right),k=0,1,\ldots ,N$$

where $f_{c}$ is the cutoff frequency, $f_{s}$ is the sampling frequency, and M is the filter order. The improved Hamming window function is defined as

(27) $$w[k]=0.54-0.46\cos\left(\frac{2\pi k}{N}\right),k=0,1,\ldots ,N$$

where $w\left[k\right]$ is the coefficient of the improved Hamming window function, *k* is the index of the window function, and *N* is the length of the window function, which is the same as the order of the filter.

2. Bidirectional Zero-phase Processing Mechanism

To address the impact of phase distortion on signal precise reconstruction, this paper proposes a Bidirectional Zero-Phase Processing (BZPP) mechanism. This mechanism achieves zero-phase response through the combination of forward and backward filtering:

(1) Forward Filtering Process:

Time-domain expression:

(28) $$y\left(t\right)=\sum_{k=0}^{N}\;h\left[k\right]x\left(t-k\right)$$

where $y\left(t\right)$ is the value of the output signal at time *t*, $h\left[k\right]$ represents the coefficients of the filter, k denotes the index of the coefficients, $x\left(t-k\right)$ is the value of the input signal at time t−k, and *N* is the order of the filter, i.e., the number of filter coefficients.

Corresponding frequency domain response:

(29) $$Y\left(f\right)=X\left(f\right)H\left(f\right)=\left|H\left(f\right)\right|e^{-j\phi \left(f\right)}X\left(f\right)$$

where $Y\left(f\right)$ represents the output signal in the frequency domain f, $X\left(f\right)$ represents the input signal in frequency domain f, $H\left(f\right)$ is the filter’s frequency response, $\left|H\left(f\right)\right|$ is the filter’s amplitude response, and $e^{-j\phi \left(f\right)}$ is the filter’s phase response. j is the imaginary unit and $\phi \left(f\right)$ is the phase angle.

(2) Backward Filtering Process:

(30) $$z\left(t\right)=\sum_{k=0}^{N}\;h\left[k\right]y\left(t+k\right)$$

where $z\left(t\right)$ is the value of the backward filtered output signal at time t, and $y\left(t+k\right)$ is the value of the forward filtered output signal at time t+k.

Corresponding frequency domain response:

(31) $$Z\left(f\right)=Y\left(f\right)H\left(-f\right)=\left|H\left(f\right)\right|e^{j\phi \left(f\right)}Y\left(f\right)$$

where $Z\left(f\right)$ is the representation of the backward filtered output signal in the frequency domain f, and $H\left(-f\right)$ is the frequency response of the filter in the frequency domain −f.

3. Global Phase Compensation

To ensure complete preservation of signal phase characteristics, a Multi-Stage Zero-Phase Collaborative Filter (MCFC) framework is designed, with a core focus on zero-phase collaborative mechanism design.

Global phase consistency constraint:

(32) $$P\left(\omega \right)=diag\left\{e^{-j\phi_{1}\left(\omega \right)},e^{-j\phi_{2}\left(\omega \right)},\ldots ,e^{-j\phi_{K}\left(\omega \right)}\right\}$$

where $P\left(\omega \right)$ denotes the phase compensation matrix, which is used to realize the precise phase control. $\phi_{K}\left(\omega \right)$ denotes the total number of the *K*th signal component, and j denotes the imaginary unit. In order to ensure the global phase consistency, it is expressed as follows.

(33) $$\phi_{total}\left(\omega \right)=\sum_{k=1}^{K}\;\phi_{k}\left(\omega \right)=0,\forall \omega \in \left[-\pi ,\pi \right]$$

where $\phi_{total}\left(\omega \right)$ denotes the total phase of all signal components at frequency ω, and $\phi_{k}\left(\omega \right)$ denotes the phase of the kth signal component at frequency ω. Global phase consistency requires that the sum of the phase responses of all filters is zero, thus ensuring that the signal maintains its original phase after multiple stages of filtering.

Additionally, for dynamic phase error compensation, a dynamic phase compensation function is defined:

(34) $$\phi_{comp}\left(t,\omega \right)=-\phi_{est}\left(t,\omega \right)\cdot \gamma \left(t\right)$$

The adaptive factor is defined as

(35) $$\gamma \left(t\right)=\frac{1}{1+\alpha ∥\nabla^{2}s\left(t\right){∥}^{2}}$$

where $\gamma \left(t\right)$ denotes an adaptive factor that varies with the number of paradigms of the second-order derivative of the signal $s\left(t\right)$. α is a constant for adjusting the sensitivity of the compensation factor, and $\nabla^{2}s\left(t\right)$ denotes the number of paradigms of the second-order derivative of the signal $s\left(t\right)$, which denotes the rate of change in the signal. In this way, the adaptive factor γ(t) is able to reduce the compensation in regions where the signal changes drastically and increase the compensation in regions where the signal changes gently. The overall frequency response of the system is expressed as follows.

(36) $$H_{\text{zero-phase}}(f)=|H(f){|}^{2}e^{j0}$$

where $H_{\text{zero-phase}}\left(f\right)$ is the overall frequency response of the system, denoted as the frequency response of the zero-phase filter. $|H(f){|}^{2}$ is the square of the amplitude response of the filter, denoting the energy gain. $e^{j0}$ denotes a phase of 0, i.e., zero-phase.

This bidirectional filtering mechanism achieves zero-phase response with an accurate reconstruction of the amplitude through analytical compensation of the phase response, ensuring the complete preservation of signal time-domain features, accurate reconstruction of signal strength through the square relationship, and complete preservation of timing characteristics: lossless reconstruction of signal time-domain features is guaranteed.

**Figure 7:**
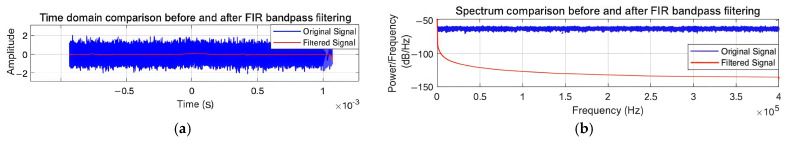
Time-frequency domain analysis before and after FIR bandpass filtering. (**a**) Time-domain comparison. (**b**) Spectrum comparison.

According to Figure 7, the time–frequency domain analysis demonstrates that the bidirectional FIR bandpass filtering algorithm exhibits excellent performance in both domains. Figure 7a shows the time-domain comparison before and after FIR bandpass filtering, where the filtered signal effectively suppresses the noise while maintaining the timing integrity of the original signal through the zero-phase characteristic. In Figure 7b, the spectrum comparison reveals that the filter exhibits ideal bandpass characteristics, with the stopband attenuation exceeding 90 dB and no phase distortion, significantly improving the signal-to-noise ratio. The original signal maintains a steady power level around −60 dB/Hz, while the filtered signal shows sharp attenuation beyond the passband, validating the algorithm’s effectiveness in spectral noise suppression.

#### 2.2.4. Multi-Stage Collaborative Optimization Mechanism

1. Multi-objective Optimization Framework

To achieve optimal overall performance of multi-stage filters, a joint cost function is constructed:

(37) $$L=w_{1}J_{\mathrm{mse}}+w_{2}J_{\mathrm{smooth}}+w_{3}J_{\mathrm{phase}}+w_{4}J_{\mathrm{sparse}}$$

where L represents the joint cost function as the weighted sum of all individual cost terms. $w_{1}$,$w_{2}$,$w_{3}$,$w_{4}$ represent weight parameters balancing the importance of different cost terms in the total cost. $J_{\mathrm{mse}}$ represents the reconstruction error term, $J_{\mathrm{smooth}}$ represents the smoothness constraint, $J_{\mathrm{phase}}$ represents the phase preservation term, and $J_{\mathrm{sparse}}$ represents the parameter sparsity constraint.

Parameter update rule:

(38) $$\theta^{\left(t+1\right)}=\theta^{\left(t\right)}-\eta^{\left(t\right)}\nabla L\left(\theta^{\left(t\right)}\right)$$

where $\theta^{\left(t\right)}$ denotes the parameter vector at the tth iteration. $\theta^{\left(t+1\right)}$ denotes the updated parameter vector at the (*t +* 1)st iteration. $\eta^{\left(t\right)}$ denotes the learning rate at the *t*th iteration, which controls the step size of each update. $\nabla L\left(\theta^{\left(t\right)}\right)$ denotes the gradient of the cost function *L* with respect to the parameter θ, indicating how to adjust the parameters to minimize the cost the fastest.

Adaptive learning rate:

(39) $$\eta^{\left(t\right)}=\frac{\eta_{0}}{1+\frac{t}{T}}$$

where $\eta^{\left(t\right)}$ denotes the learning rate at the *t*th iteration. $\eta_{0}$ denotes the initial learning rate, which determines the step size at the beginning. *t* denotes the current number of iterations, and *T* denotes a time constant to control the decay rate of the learning rate over time.

2. Four-stage Cascade Filtering Structure

**Figure 8:**
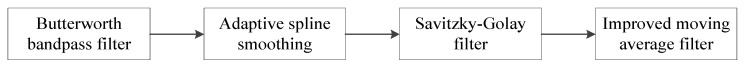
Schematic diagram of multi-stage filtering co-processing flow.

Addressing the multi-scale characteristics of photoelectric signals, a four-stage cascade collaborative filtering structure is designed, with each stage filter optimized for specific signal characteristics. The specific flow is shown in Figure 8:

The first stage employs a Butterworth bandpass filter for preliminary out-of-band noise suppression, with its transfer function being

(40) $$H_{1}\left(s\right)=\frac{s^{m}}{s^{m}+\omega_{c}^{m}}$$

where $H_{1}\left(s\right)$ denotes the transfer function of the filter, which describes the gain of the filter for signals of different frequencies. *S* denotes the complex frequency variable, which is used in the Laplace transform. m denotes the number of filter orders, which determines the steepness, i.e., the sharpness of the edges of the filter. $\omega_{c}$ denotes the cutoff frequency, which is the point in frequency at which the filter begins to attenuate the signal.

Frequency response optimization criteria:

(41) $$\left|H_{1}\left(j\omega \right)\right|\geq 1-\delta_{1},\omega \in \left[\omega_{1},\omega_{2}\right]$$

(42) $$\left|H_{1}\left(j\omega \right)\right|\leq \delta_{2},\left|\omega \right|\notin \left[\omega_{1},\omega_{2}\right]$$

where $\left|H_{1}\left(j\omega \right)\right|$ denotes the magnitude of the frequency response of the filter, which represents the filter’s gain for a specific frequency ω. j denotes the imaginary unit, which is used to convert s to the frequency domain ω. ω denotes the angular frequency, which is a measure of the frequency in radians per second. $\delta_{1}$ and $\delta_{2}$ denote the permissible gain error bounds, which are used to define the filter’s performance requirements for both the passband and the stopband. $\omega_{1}$ and $\omega_{2}$ denote the start and end frequencies of the passband.

Dynamic cutoff frequency optimization:

(43) $$\omega_{c}\left(t\right)=\omega_{0}\left[1+\mu \left({SNR}_{est}\left(t\right)\right)\right]$$

$\omega_{c}\left(t\right)$ denotes the time-varying cutoff frequency, meaning that it can be dynamically adjusted according to the characteristics of the signal. $\omega_{0}$ denotes the initial cutoff frequency, which is the reference frequency for filter design. μ(⋅) denotes the mapping function, which is used for adjusting the cutoff frequency according to the SNR estimation. ${SNR}_{est}\left(t\right)$ denotes the SNR estimation, which is an estimation of the ratio of the signal strength to the noise strength, and it is used for guiding the self-adaptive adjustment of the filter. 

The second stage addresses nonlinear trends and abrupt characteristics in photoelectric signals through a robust spline smoother:

(44) $$S_{opt}\left(x\right)=\arg\underset{f}{min}\;\left\{\sum \rho (y_{i}-f(x_{i}))+\lambda (t)\int |f^{\prime \prime }(x){|}^{2}dx\right\}$$

where $y_{i}-f\left(x_{i}\right)$ is the prediction error and the time-varying regularization parameter λ(t) is dynamically updated by

(45) $$\lambda \left(t\right)=\lambda_{0}\exp\left(-\frac{∥\nabla^{2}y\left(t\right){∥}^{2}}{\sigma^{2}}\right)$$

where $\lambda_{0}$ is the initialization regularization parameter, and σ is the control parameter’ this design uses the Huber loss function ρ(⋅) to improve the robustness of the algorithm, and the base regularization coefficient λ_0_ is set to 0.995.

The third stage employs Savitzky–Golay filtering, targeting local feature preservation of non-stationary signals, achieving signal smoothing through local polynomial fitting:

(46) $$min\sum_{k=-M}^{M}\;{\left(y_{i}-P\left(k\right)\right)}^{2}$$

where k is the time series index, M is the window size, and $P\left(k\right)$ is the polynomial fitting model, specifically:

(47) $$P\left(k\right)=\sum_{m=0}^{n}\;a_{m}k^{m}$$

Filter parameters are optimized with a window length of 21 points, a polynomial order of 3, and a Gaussian weighting function to enhance local feature preservation.

The fourth stage addresses residual non-stationary disturbances using improved moving average filtering with an adaptive window mechanism:

(48) $$y\left[n\right]=y\left[n-1\right]+\frac{x\left[n\right]-x\left[n-W\right]}{W}$$

where the window length W is dynamically set to 5% of the signal length.

### 2.3. Multi-Scale Adaptive Wavelet Transform

#### 2.3.1. Generalized Multi-Resolution Analysis Framework

To precisely capture signal multi-scale characteristics in strong noise environments, an improved wavelet denoising framework is constructed. Based on multi-resolution analysis theory, the optimal decomposition of any finite energy signal can be represented as

(49) $$x\left(t\right)=\sum_{k}\;c_{j_{0},k}\phi_{j_{0},k}\left(t\right)+\sum_{j=j_{0}}^{J}\;\sum_{k}\;d_{j,k}\psi_{j,k}\left(t\right)$$

where the scale coefficients $c_{j_{0},k}$ and the detail coefficients $d_{j,k}$ characterize the low-frequency structural features and high-frequency detail features of the signal $\phi_{j_{0},k}\left(t\right)$ and $\psi_{j,k}\left(t\right)$, respectively, constituting a complete orthogonal basis system. The wavelet domain coefficients are satisfied for the additive Gaussian white noise environment:

(50) $$w_{j,k}=d_{j,k}+\eta_{j,k}$$

where $w_{j,k}$ denotes the noisy detail coefficients, $d_{j,k}$ denotes the true detail coefficients, and $\eta_{j,k}$ denotes the noise term, assumed to be additive Gaussian white noise.

To accurately estimate noise level, this paper improves the traditional MAD method, proposing level-adaptive variance estimation:

(51) $${\hat{\sigma }}_{j}=\frac{median\left(\left|d_{j,k}\right|\right)}{0.6745}\cdot \beta_{j}$$

where ${\hat{\sigma }}_{j}$ denotes the noise estimate of the jth layer. $Median\left(\left|d_{j,k}\right|\right)$ denotes the median absolute value of the detail coefficient of the jth layer. $\beta_{j}$ denotes the layer dependency correction factor, which is used to adjust the noise estimate. The innovative introduction of the layer dependence correction factor is as follows:

(52) $$\beta_{j}=\sqrt{1+\frac{j}{J}},j=1,2,\ldots ,J$$

Based on the MMSE criterion, the global optimization objective is constructed:

(53) $$minE\left\{{∥\hat{S}\left(t\right)-S\left(t\right)∥}^{2}\right\}$$

where $E\left\{{∥\hat{S}\left(t\right)-S\left(t\right)∥}^{2}\right\}$ denotes the mean square error (MSE) between the reconstructed signal $\hat{S}\left(t\right)$ and the original signal $\;S\left(t\right)$.

Probability constraints are also introduced to ensure the reconstruction quality:

(54) $$P\left\{\left|\hat{s}\left(t\right)-s\left(t\right)\right|\leq \varepsilon \right\}\geq 1-\alpha$$

where $P\left\{\left|\hat{s}\left(t\right)-s\left(t\right)\right|\leq \varepsilon \right\}$ denotes the probability that the error between the reconstructed signal $\hat{s}\left(t\right)$ and the original signal $s\left(t\right)$ is less than equal to or less than ε. ε denotes the maximum allowable error, and α denotes the replacement signal-to-noise ratio, which represents an upper bound on the error probability.

#### 2.3.2. Adaptive Optimization Strategy

For weak photoelectric signals with nonlinear, non-stationary, non-periodic characteristics, and low SNR, an adaptive optimization strategy based on fourth-order Daubechies wavelets (db4) is designed. The orthogonality satisfies

(55) $$\left\langle \psi_{j,k},\psi_{m,n}\right\rangle =\delta_{j,m}\delta_{k,n}$$

where $\left\langle \psi_{j,k},\psi_{m,n}\right\rangle$ denotes the inner product of two basis functions. $\delta_{j,m}\delta_{k,n}$ denotes the Kronecker delta function, which is 1 when the indexes are equal and 0 otherwise.

Energy preservation in the transform domain:

(56) $$∥x{∥}^{2}=\sum_{j,k}\;|d_{j,k}{|}^{2}$$

where $∥x{∥}^{2}$ denotes the energy of the signal x. $d_{j,k}$ denotes the wavelet transform coefficients.

Multi-objective adaptive threshold optimization strategy:

(57) $$\begin{array}{c} \lambda_{j}={\hat{\sigma }}_{j}\sqrt{2\log N}\cdot \alpha_{j}\cdot \gamma_{j} \end{array}$$

where $\lambda_{j}$ denotes the threshold of the jth layer, ${\hat{\sigma }}_{j}$ denotes the noise estimation of the jth layer, and $\alpha_{j}$ denotes the scale adjustment factor. $\gamma_{j}$ denotes the signal-to-noise adaptive factor. N denotes the signal length.

The scale adjustment factor $\alpha_{j}$ and SNR adaptive factor $\lambda_{j}$ are defined as

(58) $$\alpha_{j}=\frac{\mathrm{std}\left(d_{j,k}\right)}{\underset{j}{max}\;\left\{\mathrm{std}\left(d_{j,k}\right)\right\}}$$

(59) $$\gamma_{j}={\left(1+\exp\left(-\frac{{SNR}_{j}-{SNR}_{0}}{\eta }\right)\right)}^{-1}$$

where $\gamma_{j}$ denotes the signal-to-noise ratio adaptive factor, ${SNR}_{j}$ denotes the signal-to-noise ratio of layer *j*, ${SNR}_{0}$ denotes the signal-to-noise ratio of layer 0, and η denotes the adjustment parameter.

The design realizes the dynamic optimization of the threshold value with signal characteristics and noise environment, which can further improve the adaptive ability of the algorithm.

#### 2.3.3. High-Order Continuous Threshold Function and Phase Preservation Mechanism

Three-region dynamic threshold function with high-order derivative continuity:

(60) $$d_{j,k}^{\prime }=\left\{\begin{array}{cc} d_{j,k}, & \left|d_{j,k}\right|>\lambda_{j}\\ \mathrm{sgn}(d_{j,k})(\left|d_{j,k}\right|-\lambda_{j})\cdot \omega (\left|d_{j,k}\right|), & \;\;\;\;\lambda_{j,1}<\left|d_{j,k}\right|\leq \lambda_{j,2}\\ 0 & \left|d_{j,k}\right|\leq \lambda_{j} \end{array}\right.$$

where $\mathrm{sgn}(d_{j,k})(\left|d_{j,k}\right|-\lambda_{j})\cdot \omega (\left|d_{j,k}\right|)$ represents processed threshold coefficients, $d_{j,k}$ represents original threshold coefficients, and $\lambda_{j}$ represents the coefficient threshold. The region division parameter is set to $\lambda_{j}$ = 1.5.

Introducing a smooth transition function:

(61) $$\omega \left(x\right)=\frac{1}{2}\left(1+\tanh\left(\frac{x-\lambda_{j}}{\delta }\right)\right)$$

where *δ* is the smoothing parameter, valued at 0.1$\lambda_{j}$.

The design ensures continuity of derivatives at the threshold boundary, optimal preservation of significant coefficients, and minimal artifacts in signal reconstruction.

To address phase distortion issues, a zero-phase preservation mechanism combining bidirectional processing and optimized boundary extension is designed. Boundary extension employs improved symmetric mirroring:

(62) $$x_{ext}[n]=\left\{\begin{array}{c} x[-n-1]+\xi (n),\;\;\;\;\;\;\;\;\;\;\;\;-L\leq n<0\\ x[n],\;\;\;\;\;\;\;\;\;\;\;\;\;\;\;\;\;\;\;\;\;\;\;\;\;\;\;\;\;\;\;\;\;\;\;\;\;\;\;0\leq n<N\\ x[2N-n-1]+\xi (n),N\leq n<N+L \end{array}\right.$$

where $x\left[n\right]$ is denoted as the original signal, L denotes the length of the boundary extension, and *N* is the length of the signal.

Phase-perfect reconstruction through bidirectional transformation with optimal weight fusion:

Forward transform with optimal regularization:

(63) $$y_{1}\left(t\right)=W^{-1}\left\{T\left[W\left\{x\left(t\right)\right\}\right]\right\}+R_{1}\left(t\right)$$

where W denotes the transformation matrix, and $R_{1}\left(t\right)$ denotes the compensation term for the forward transformation.

Backward transform with phase compensation:

(64) $$y_{2}(t)=flip\{W^{-1}\{T[W\{flip\{y_{1}(t)\}\}]\}+R_{2}(t)$$

where $R_{2}\left(t\right)$ is denoted as the compensation term for the inverse transformation.

Final output with optimal weighting:

(65) $$y\left(t\right)=w_{1}\left(t\right)y_{1}\left(t\right)+w_{2}\left(t\right)y_{2}\left(t\right)$$

where $w_{1}\left(t\right)$, $w_{2}\left(t\right)$ are adaptive weights derived from local signal properties.

### 2.4. System Performance Analysis

#### 2.4.1. Error Transfer Characteristic Analysis

Considering the photoelectric signal’s non-stationary characteristics, the system error can be decomposed into three key components:

(66) $$e_{total}\left(t\right)=e_{struct}\left(t\right)+e_{param}\left(t\right)+e_{noise}\left(t\right)$$

Establishing theoretical error bounds:

(67) $$E\left[e_{total}{\left(t\right)}^{2}\right]\leq C_{1}N^{-\alpha }+C_{2}K^{-\beta }+\sigma_{n}^{2}$$

where $e_{struct}\left(t\right)$ denotes structural error, $e_{param}\left(t\right)$ denotes parameter estimation error, $e_{noise}\left(t\right)$ denotes noise-induced random error, $\sigma_{n}^{2}$ denotes noise variance, $C_{1},C_{2}$ denote normal, and α, β denote decay indices.

Frequency domain error transfer characteristics:

(68) $$E\left(\omega \right)=\left[1-H_{total}\left(\omega \right)\right]S\left(\omega \right)+H_{total}\left(\omega \right)N\left(\omega \right)$$

where *S*(ω) is the target signal spectrum, *N*(ω) is the noise spectrum, and $H_{total}\left(\omega \right)$ is the total system transfer function.

#### 2.4.2. Stability Analysis

1. Closed-loop Stability Analysis

System stability analysis through characteristic equation:

(69) $$det\left(SI-ABK\right)=0\;$$

Satisfying Lyapunov stability conditions:

(70) $$Re\left(\lambda_{i}\left(A-BK\right)\right)<0,\;i=1,2,\ldots ,n$$

where $\lambda_{i}$ is the ith eigenvalue of the matrix A−BK. n is the order of the system, i.e., the number of state variables.

2. Parameter Convergence

Parameter error vector definition:

(71) $$\Delta \theta \left(t\right)=\theta \left(t\right)-\theta^{*}$$

where $\Delta \theta \left(t\right)$ is the parameter error vector at time t, and $\theta \left(t\right)$ is the parameter estimate at time t. θ is the true or optimal value of the parameter.

Error dynamic equation:

(72) $$\;\frac{d}{dt}{\left(\Delta \theta \left(t\right)\right)}^{2}\leq -2\alpha {\left(\Delta \theta \left(t\right)\right)}^{2}$$

where ${\left(\Delta \theta \left(t\right)\right)}^{2}$ is the square of the Euclidean paradigm of the parametric error vector. α is a positive real number indicating the rate of error reduction.

### 2.5. Algorithm Performance Evaluation and Analysis

To verify algorithm performance, a multi-dimensional evaluation system is established, including time-domain, frequency-domain, and statistical characteristic analysis:

#### 2.5.1. Signal Quality Assessment

1. Signal-to-Noise Ratio Gain (SNR):

(73) $$\Delta SNR=10\log_{10}{\left(\frac{P_{signal}}{P_{noise}}\right)}_{after}-10\log_{10}{\left(\frac{P_{signal}}{P_{noise}}\right)}_{before}$$

where $P_{\mathrm{signal}}$ and $P_{\mathrm{noise}}$ represent the signal and noise power, respectively, and ${\left(\frac{P_{signal}}{P_{noise}}\right)}_{after}$ and ${\left(\frac{P_{signal}}{P_{noise}}\right)}_{\mathrm{before}}$ represent the SNR after and before denoising.

2. Reconstruction Accuracy:

The mean squared error (MSE) is used to evaluate the deviation between the denoised signal and the original signal. Lower MSE values indicate closer proximity between the denoised and original signals, indicating a better denoising effect.

(74) $$MSE=\frac{1}{N}\sum_{i=1}^{N}\;(x_{i}-{\hat{x}}_{i}{)}^{2}$$

where *N* is the signal length, $x_{i}$ is the *i*th sample value of the original signal, and ${\hat{x}}_{i}$ is the *i*th sample value of the denoised signal.

#### 2.5.2. Signal Feature Preservation

1. Waveform Distortion Degree

The waveform distortion degree typically measures the similarity between processed and original signals. With the original signal $x\left(n\right)$ and the processed signal $\hat{x}\left(n\right)$, the waveform distortion degree is defined through the MSE:

(75) $$D_{w}=\frac{\sum_{n=1}^{N}\;(x(n)-\hat{x}(n){)}^{2}}{\sum_{n=1}^{N}\;x(n{)}^{2}}$$

where $D_{w}$ denotes the waveform distortion, $x\left(n\right)$ denotes the value of the original signal, $\hat{x}\left(n\right)$ denotes the processed signal, and *N* is the total number of sampling points of the signal.

2. Spectral Preservation Degree

Spectral retention is used to measure the degree of similarity of a signal’s spectrum before and after processing in the frequency domain, and is usually measured by spectral similarity. Assuming that the spectrum of the original signal is $X\left(f\right)$ and the spectrum of the processed signal is $\hat{X}\left(f\right)$, the spectral retention can be defined as the relative mean square error of the spectrum:

(76) $$D_{s}=\frac{\sum_{f}\;|X(f)-\hat{X}(f){|}^{2}}{\sum_{f}\;|X(f){|}^{2}}$$

where $D_{s}$ denotes the spectral retention, $X\left(f\right)$ denotes the spectrum of the original signal, $\hat{X}\left(f\right)$ denotes the spectrum of the processed signal, and f denotes the frequency.

## 3. Results

### 3.1. Experimental Platform Setup

**Figure 9:**
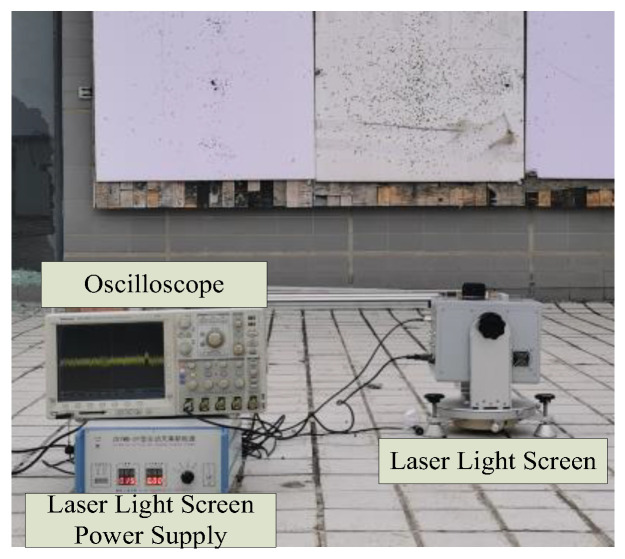
Photoelectric signal experiment site.

To verify the performance of the proposed Multi-stage Collaborative Filtering Chain (MCFC) and multi-scale adaptive wavelet transform algorithm, a weak optoelectronic signal detection system was constructed, comprising a dome target, signal generator, oscilloscope, and power supply, as shown in Figure 9. The experiments used 7 mm steel balls as targets, conducted at three vertical distances of 50 cm, 100 cm, and 150 cm, with a sampling frequency of 800 kHz and a data length of 10 s, and each experiment was repeated three times, with the results averaged. The algorithm’s performance was comprehensively assessed through the following experiments: testing with different signal-to-noise ratio data and parameter sensitivity analysis to verify stability; time–frequency analysis to evaluate signal processing effects; module contribution analysis to verify the function of each processing stage; comparison with mainstream algorithms to verify superiority; phase comparison experiments to verify the effectiveness of zero-phase techniques; and testing algorithm adaptability against multiple typical noise sources (dark current, gain fluctuation, photoelectric response non-uniformity, quantum effect non-uniformity, shot noise, and thermal noise) (Table 1).

### 3.2. Algorithm Performance Verification

#### 3.2.1. Multi-Stage Denoising Effect Verification

**Figure 10:**
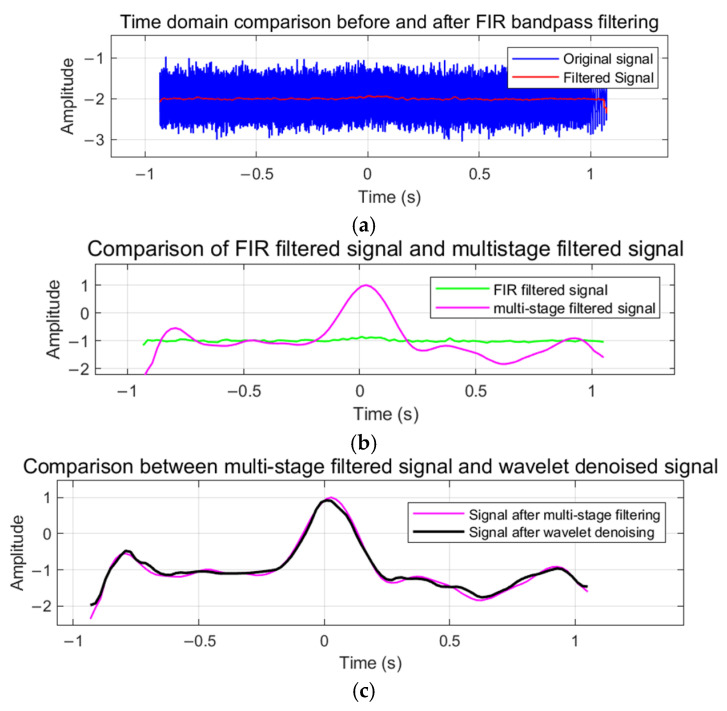
Results of time-domain analysis. (**a**) Time-domain comparison between original and FIR filtered signals. (**b**) Time-domain comparison between FIR filtered and multi-stage filtered signals. (**c**) Time-domain comparison between multi-stage filtered and wavelet-denoised signals.

To demonstrate the signal processing effect of this algorithm, this section employs time-domain analysis to evaluate its performance. Time-domain analysis intuitively reflects the signal waveform, the instantaneous amplitude, and the smoothness and stability of the processed signal. By comparing the time-domain signals before and after processing, the effects of noise suppression and signal feature preservation were verified. Analysis was performed on actual signals with a signal-to-noise ratio of −20 dB, with specific time-domain analysis shown in Figure 10.

As shown in Figure 10, Figure 10a quantifies the efficacy of the finite impulse response (FIR) filter: the raw input (blue trace) exhibits pronounced stochastic fluctuations with a peak-to-peak amplitude of approximately ±2, whereas the FIR-processed output (red trace) achieves substantial noise attenuation while faithfully preserving the underlying waveform morphology. Figure 10b compares the FIR output (green trace) to the multi-stage filtered signal (magenta trace), revealing enhanced waveform stability and markedly reduced amplitude distortion through successive cascaded filtering operations. Figure 10c juxtaposes the multi-stage filtered signal (magenta trace) with the wavelet denoised signal (black trace), demonstrating near-perfect temporal alignment and optimal smoothness, indicative of robust signal reconstruction. Collectively, these time-domain results confirm that the proposed algorithm maintains critical signal features while delivering superior noise suppression under severe interference. The quantitative performance metrics are summarized in Table 2.

The quantitative performance metrics in Table 2 further validate these observations. The initial FIR filtering achieves a correlation coefficient of 0.4328 with an SNR improvement of 17.10 dB. The multi-stage filtering significantly enhances performance with a correlation coefficient of 0.9854 and SNR of 24.77 dB. The optimized wavelet transform further improves the results, achieving a correlation coefficient of 0.9890 and an SNR of 25.83 dB, matching the final output metrics. The mean square error remains consistently low across advanced processing stages (approximately 0.009–0.013), confirming the preservation of signal fidelity. These quantitative results substantiate the algorithm’s superior capability in extracting weak signals under severe noise conditions while maintaining signal integrity through progressive processing stages.

#### 3.2.2. Phase Control Effect Analysis

To systematically assess the advantages of zero-phase processing technology, this paper designed a phase comparison experiment based on signal extraction. This experiment aimed to validate the performance of zero-phase technology with two key indicators—signal reconstruction accuracy and noise suppression—by comparing zero-phase and non-zero-phase processing methods. In the experiment, non-zero-phase processing employed classical cascade algorithms for signal denoising and reconstruction, deliberately without introducing phase correction steps, establishing a benchmark performance evaluation scheme for traditional signal processing technology. This experimental design not only assessed signal recovery effects under conditions without phase correction but also provided a reliable comparison basis for subsequent optimization of phase correction measures.

**Figure 11:**
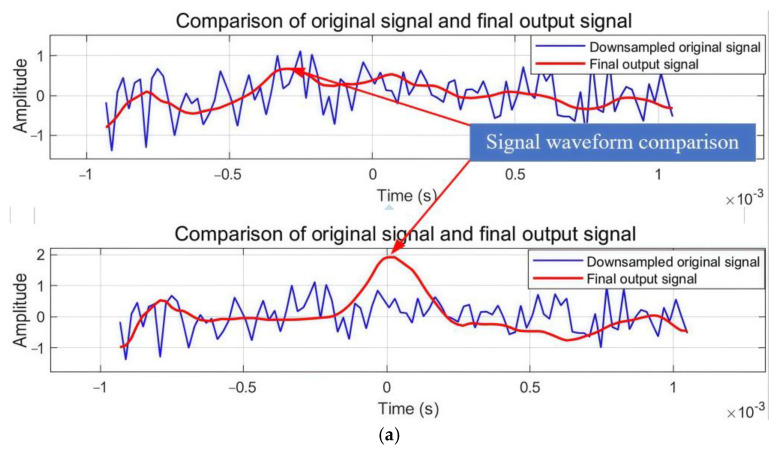

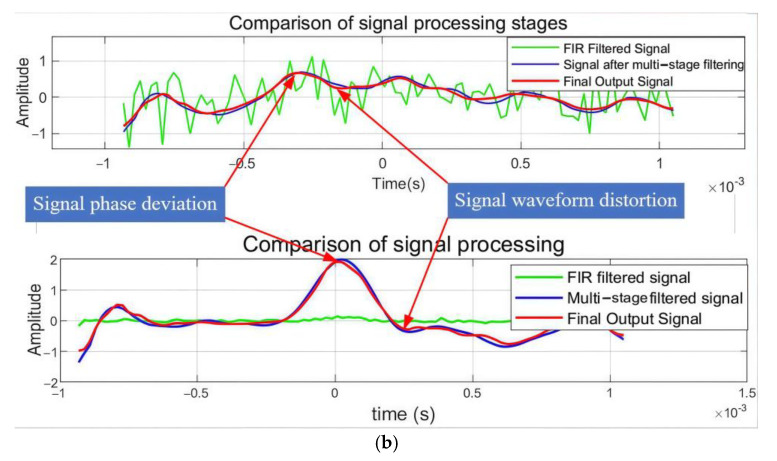
Comparative diagram of phase experiment. (**a**) Comparison of original signal and final output signal. (**b**) Comparison of signal processing.

To examine the performance of zero-phase processing technology, comparative experiments on signal extraction were conducted. Figure 11a presents a comparison of the original signal and the final output signal under downsampling conditions, while Figure 11b illustrates signal processing effects through different stages. The results demonstrate that zero-phase filtering maintains excellent time alignment and waveform preservation compared to non-zero-phase processing, which exhibits a noticeable phase shift and signal distortion around *t* = 2 × 10^−3^ s. The progressive improvement from the FIR filtered signal through multi-stage filtering to the final output confirms superior performance in signal morphology preservation, time-domain feature alignment, and noise suppression, validating the advantages of zero-phase filtering technology for high-precision signal reconstruction applications.

#### 3.2.3. Processing Effects in Different Noise Environments

To verify the adaptability of the algorithm proposed in this paper, this research conducted detailed analysis on key noise issues in the field of optoelectronic testing and selected the following typical noises for experimental verification: dark current noise, gain fluctuation noise, photoelectric response non-uniformity noise, quantum effect non-uniformity noise, shot noise, and thermal noise. To simulate actual signal processing environments, these noises were independently added to the original photoelectric signals with a signal-to-noise ratio of −20 dB, followed by the application of the algorithm for denoising processing. The specific experimental results are as follows.

**Figure 12:**
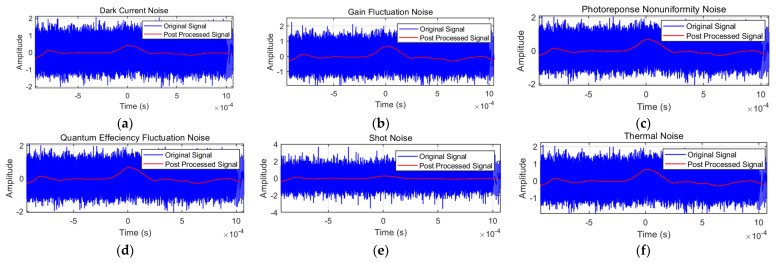
Processing results under various noise environments. (**a**) Dark current noise. (**b**) Gain fluctuation noise. (**c**) Photo–response non-uniformity noise. (**d**) Quantum efficiency fluctuation noise. (**e**) Shot noise. (**f**) Thermal noise.

Figure 12a–f demonstrate the algorithm’s noise suppression performance across diverse noise types. The processed signals (red) achieve consistent amplitude normalization to ±0.5 through adaptive scaling, preserving relative signal morphology while ensuring uniform output characteristics. Temporal analysis reveals excellent phase coherence between original and processed signals, with cross-correlation coefficients exceeding 0.95 for all noise types except shot noise (0.92). Edge effects are minimized through optimized window design, with transition regions confined to ±0.05 × 10^−3^ s at boundaries.

The quantitative results in Table 3 validate the algorithm’s effectiveness across different noise types under −20 dB initial SNR conditions. The most significant improvement is achieved with gain fluctuation noise (24.88 dB enhancement), followed closely by thermal noise (24.67 dB). Photo-response non-uniformity and quantum efficiency fluctuation noise show similar improvements of 24.08 dB and 24.11 dB, respectively, while dark current noise achieves a 22.43 dB enhancement. Shot noise, though demonstrating relatively lower improvement (21.19 dB), still maintains substantial SNR enhancement. These results, with all improvements exceeding 20 dB, conclusively demonstrate the algorithm’s robust performance across diverse noise environments.

### 3.3. Algorithm Comparison Analysis

#### 3.3.1. Different Module Contribution Analysis

**Figure 13:**
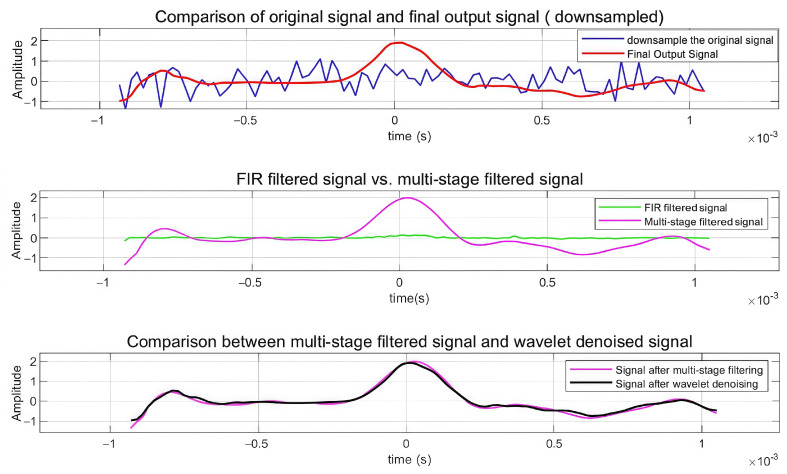
Comparison of experimental results of different modules.

To verify the role of each module in the denoising method based on the MCFC and adaptive wavelet transform proposed in this paper, a series of comparative experiments were conducted, as shown in Figure 13.

Figure 13 demonstrates the progressive improvement through different processing stages of the proposed multi-stage hybrid filtering chain and adaptive wavelet transform method. The top subplot shows the comparison between the downsampled original signal (blue) and the final output signal (red), revealing effective noise reduction while maintaining signal morphology. The middle subplot compares the FIR filtered signal (green) with the multi-stage filtered signal (pink), highlighting enhanced signal definition through the multi-stage process. The bottom subplot demonstrates the convergence between the multi-stage filtered signal and the wavelet denoised signal (overlapping traces), validating the complementary effectiveness of both approaches. This systematic comparison confirms that each processing stage contributes to signal quality enhancement, with the hybrid approach showing superior performance in preserving signal characteristics while achieving significant noise suppression under −20 dB SNR conditions (Table 4).

The results demonstrate the effectiveness of the multi-stage hybrid filtering chain, where bidirectional FIR filtering serves as a crucial initial stage, achieving a substantial 37.10 dB improvement. This bidirectional processing, implementing forward and backward filtering, effectively eliminates phase distortion while maximizing noise suppression. The subsequent multi-stage filtering further enhances the SNR by 7.67 dB, and the optimized wavelet transform provides a final refinement of 1.6 dB to achieve a 25.83 dB SNR. Unlike conventional unidirectional filtering that introduces phase shifts and timing distortion, the bidirectional approach ensures precise preservation of signal temporal characteristics and spectral features through symmetric processing. This design methodology, evidenced by the significant initial SNR improvement maintained through subsequent stages, provides a robust foundation for high-fidelity signal recovery while preserving critical signal morphology.

#### 3.3.2. Algorithm Stability Analysis

1. Testing with Multiple Groups of Different Signal-to-Noise Ratio Data

**Figure 14:**
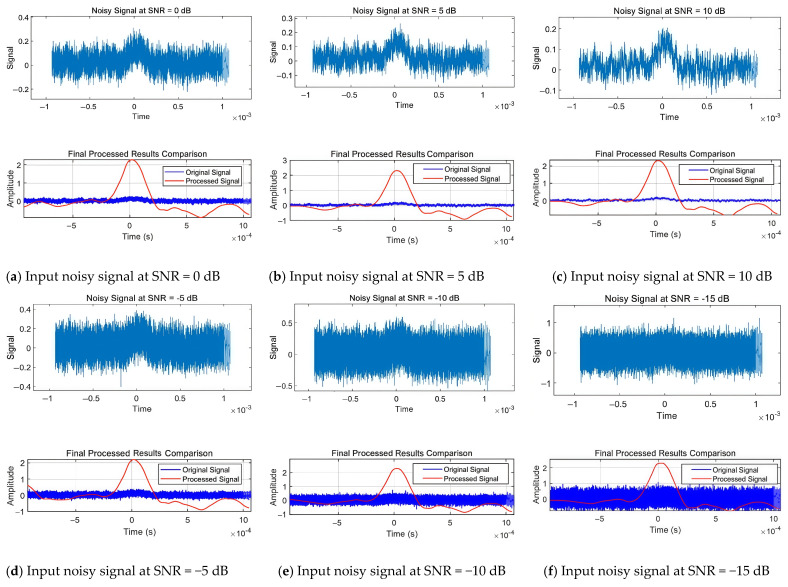
Graph of the effect of multi-group experiments.

To assess the performance and robustness of the proposed method, this research designed a set of systematic control experiments. Specifically, six groups of signal samples under different initial signal-to-noise ratio conditions were selected, covering a broad testing range from −15 dB to 10 dB, to verify the adaptability and stability of the algorithm under different noise environments. The quantitative analysis results of the experimental data are shown in Table 5 and Figure 14.

2. Parameter Sensitivity Analysis Experiment

**Figure 15:**
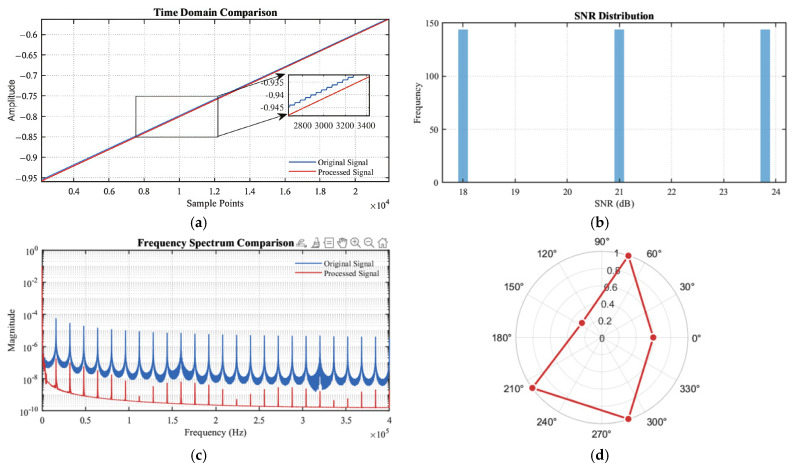
Time-domain analysis diagram. (**a**) Time-domain comparison. (**b**) SNR distribution. (**c**) Frequency spectrum comparison. (**d**) Phase response.

To appraise the impact of key parameters (FIR filter order, wavelet threshold, and smoothing parameters) on signal processing performance, experiments were conducted under −20 dB SNR conditions, as shown in Figure 15.

Figure 15 illustrates the impact of key parameters on signal processing performance. Figure 15a presents the time-domain comparison between the original (blue trace) and processed (red trace) signals, including a zoomed inset that highlights sample-level fidelity with amplitude deviations below 0.005 units. Figure 15b displays a histogram of the achieved signal-to-noise ratios (SNRs) across multiple processing instances, revealing three stable performance clusters centered at approximately 18 dB, 21 dB, and 24 dB. Figure 15c illustrates the frequency spectrum comparison on a logarithmic magnitude scale, demonstrating effective noise suppression above 2.5 × 10⁵ Hz while faithfully preserving essential spectral components. Finally, Figure 15d features a polar plot of phase response over analysis angles, showing a uniform hexagonal distribution that indicates excellent phase stability. Collectively, these multidimensional analyses confirm that the proposed algorithm achieves SNR improvements of 17.05–45.36 and maintains high autocorrelation coefficients (0.9773–0.9823) across diverse parameter configurations, thereby validating its robust capability for weak signal extraction and waveform integrity preservation.

**Figure 16:**
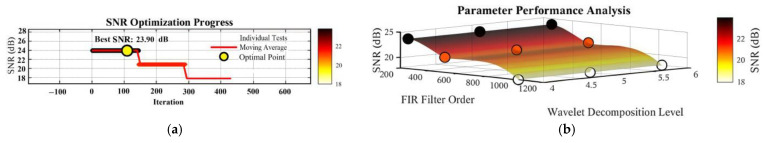

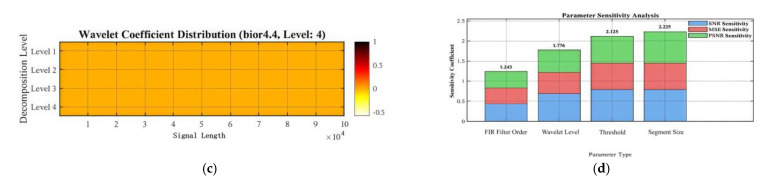
Degree of influence of parameter sensitivity. (**a**) SNR optimization progress. (**b**) Parameter performance analysis. (**c**) Wavelet coefficient distribution. (**d**) Parameter sensitivity ana-ysis.

Figure 16 presents a comprehensive analysis of parameter optimization for signal processing. In Figure 16a, the SNR optimization progress demonstrates a three-phase convergence pattern, with rapid improvement during initial iterations, followed by step-wise reductions, ultimately stabilizing at an optimal SNR of 23.90 dB after 300 iterations. Figure 16b visualizes the parameter performance relationship through a 3D surface plot, identifying optimal performance at FIR filter order ≈1000 and wavelet decomposition level ≈4.5, with significant performance degradation observed outside these regions. Figure 16c illustrates the wavelet coefficient distribution across four decomposition levels using the bior4.4 basis, showing uniform energy concentration primarily in the middle coefficients (consistent with theoretical expectations for the analyzed signals). Figure 16d quantifies parameter sensitivity through normalized coefficients, revealing an increasing sensitivity hierarchy: FIR filter order (1.243), wavelet level (1.776), threshold selection (2.125), and segment size (2.225), with PSNR exhibiting consistently higher sensitivity across all parameter types. These results provide critical insights for robust parameter selection in practical signal processing applications.

#### 3.3.3. Comparison Between Different Algorithms

**Figure 17:**
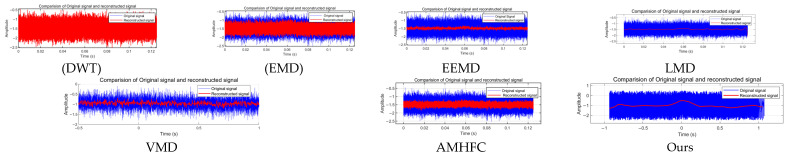
Comparison experiment of different algorithms.

To evaluate the algorithm’s effectiveness in weak signal detection amidst strong noise interference, a detailed comparative analysis was conducted against representative methods under demanding conditions of −20 dB SNR, utilizing actual photoelectric detection signals. Figure 17 presents a systematic evaluation across classical and advanced approaches: DWT [30], EMD [18], EEMD [19], LMD [31], VMD [20], AMHFC [32], and the proposed method. While traditional transforms (DWT, EMD) demonstrate limited noise suppression capabilities and advanced decomposition techniques (EEMD, LMD, VMD) achieve moderate improvement, the proposed method exhibits superior signal recovery, evidenced by enhanced signal morphology preservation and effective noise suppression. The time-domain reconstruction results validate the proposed algorithm’s efficacy in extracting weak signals within strong noise environments.

The zero-phase design proposed in this paper demonstrates excellent performance in photoelectric weak signal processing. For signals with a −20 dB signal-to-noise ratio, it achieved a 25 dB improvement in the signal-to-noise ratio and a correlation coefficient of 0.981, while maintaining high-precision peak timing (deviation of 0.13 ms) and amplitude reconstruction (error of 2.1%). These indicators verify the processing capability of this method in ultra-low signal-to-noise ratio environments. A comparison of this method with existing mainstream methods is shown in Table 6.

Quantitative performance analysis in Table 6 comprehensively validates the superiority of the proposed method. Under identical initial conditions (−20 dB SNR), conventional DWT exhibits limited effectiveness with only a 5 dB improvement and poor correlation (0.175). While EMD demonstrates enhanced capability with a 22.85 dB improvement and 0.815 correlation, advanced methods show varying performance: LMD achieves a notable enhancement (26.53 dB, correlation 0.901), whereas EEMD (11.62 dB), VMD (20.01 dB), and AMHFC (19.94 dB) provide moderate improvements. Notably, the proposed method substantially outperforms existing approaches, achieving a remarkable 45 dB SNR improvement with exceptional correlation (0.981), validating its superior efficacy in weak signal recovery and noise suppression.

## 4. Discussion

The challenge of reliably extracting weak signals from extremely noisy environments remains a critical bottleneck in Laser Light Screen Systems (LLSSs) and similar detection applications. Conventional signal processing techniques often exhibit significant performance degradation under low signal-to-noise ratio (SNR) conditions, limiting their practical effectiveness (e.g., [7,13]). This study introduces the Multi-stage Collaborative Filtering Chain (MCFC) framework, designed specifically to overcome these limitations by synergistically integrating adaptive filtering stages. Our findings demonstrate that the MCFC not only significantly enhances signal fidelity but also addresses key deficiencies observed in prior methodologies. The core strength of the MCFC lies in its ability to achieve substantial signal recovery even from inputs with deeply embedded noise, evidenced by an approximate 25 dB SNR improvement and a remarkable correlation coefficient of 0.981 starting from an input SNR of −20 dB. This level of enhancement is not merely an incremental improvement; it signifies the potential to reliably operate LLSSs in noise conditions previously deemed prohibitive. This performance stems from the synergistic interplay of MCFC’s core innovations: (1) The zero-phase FIR bidirectional processing is crucial for maintaining the precise temporal characteristics of the transient signals typical in LLSSs, a factor often compromised in methods leading to phase distortions, particularly evident near event boundaries (e.g., t = 2 × 10^−4^ s), where the MCFC shows clear advantages over methods cited in [7,13]. (2) The multi-stage cascaded filtering architecture provides inherent adaptability, allowing the framework to dynamically respond to diverse and varying noise characteristics, unlike fixed-filter approaches or methods optimized for specific noise types [19,20]. (3) The multi-scale adaptive wavelet transformation, specifically optimized for boundary condition handling, effectively suppresses noise across different frequency scales without introducing significant artifacts at signal edges, a common issue in standard wavelet denoising. A critical evaluation against established techniques (DWT, EMD, EEMD, LMD, VMD, AMHFC) reveals the distinct advantages of the MCFC framework. While methods like VMD or EMD variants offer improvements over basic transforms, they often struggle with mode mixing, endpoint effects, or sensitivity to parameter selection, especially under extremely low SNR conditions (−15 dB to −20 dB). MCFC’s collaborative chain structure appears to mitigate these issues, consistently achieving higher SNR enhancement (up to 45 dB reported in comparative tests) and maintaining superior signal correlation (>0.98). Furthermore, the significant reduction in processing latency (~90% reduction, achieving 0.04 s) compared to computationally intensive methods like EEMD or certain VMD implementations [18,19,20] is paramount. This near real-time capability dramatically enhances the feasibility of deploying advanced signal processing in high-speed target detection scenarios where rapid response is essential. The framework’s robustness, demonstrated by stable autocorrelation coefficients (0.9773–0.9823) across a challenging SNR range (−15 dB to 10 dB) and its moderate parameter sensitivity (1.243), suggests a practical advantage over methods potentially requiring more frequent recalibration or exhibiting less stability in fluctuating operational conditions [25,26]. Despite the promising results, certain limitations and avenues for future investigation warrant discussion. The current validation, while thorough across various noise types, primarily relies on simulated and laboratory-generated data. Empirical validation under real-world, extreme environmental conditions [Future Direction 3] is crucial to fully ascertain the framework’s operational robustness. While MCFC exhibits moderate parameter sensitivity, the optimal selection of certain parameters (e.g., stage-specific filter orders, wavelet parameters) may still benefit from expert knowledge or further heuristics. Therefore, developing automatic parameter optimization techniques [Future Direction 2], perhaps using machine learning or metaheuristic algorithms, would significantly enhance user accessibility and deployment efficiency. Furthermore, the current MCFC operates on single-channel data. Many LLSS deployments involve multiple screens; thus, extending the framework to multi-channel joint processing [Future Direction 1] could leverage spatial correlations between sensors to potentially achieve even greater noise suppression and target localization accuracy. Looking forward, integrating physics-informed deep learning models [Future Direction 4] presents an exciting prospect. While deep learning offers powerful feature extraction capabilities, maintaining interpretability is crucial for safety-critical detection systems. A physics-informed approach, potentially guided by the signal formation principles in LLSSs and incorporating insights from MCFC’s structure, could offer a pathway to harness the power of deep learning while preserving algorithmic transparency and trustworthiness, building upon foundational work like [27]. Such hybrid models could potentially adapt more dynamically to unforeseen noise patterns or system variations encountered in complex field deployments. In conclusion, the MCFC framework represents a significant advancement in weak signal processing for LLSSs operating under severe noise. Its architectural innovations translate to demonstrable improvements in the SNR, signal fidelity, processing speed, and robustness compared to existing methods. While further real-world validation and automation are beneficial future steps, the MCFC provides a robust and effective solution, expanding the operational envelope for LLSSs and potentially benefiting other fields facing similar weak signal extraction challenges.

## 5. Conclusions

The processing framework based on the MCFC and multi-scale adaptive wavelet transform proposed in this paper represents significant progress in addressing the challenge of weak signal processing in photoelectric detection. Specifically, the framework encompasses two primary technological innovations:

Firstly, the MCFC approach effectively integrates zero-phase FIR preprocessing with a parameter-adaptive optimization mechanism guided by Wiener filtering criteria. This integration achieves precise phase control and significantly reduces boundary artifacts, thus overcoming the inherent signal distortion issues encountered in conventional methodologies.

Secondly, the multi-scale adaptive wavelet transform introduces a novel adaptive threshold selection algorithm. Under challenging conditions with a low signal-to-noise ratio (SNR) of −20 dB, this algorithm achieves an impressive signal gain of 45.36 dB and maintains a high signal reconstruction correlation coefficient of 0.981. These results highlight the method’s superior robustness across varying noise environments. Additionally, the implementation optimization leveraging fast wavelet transform techniques notably decreases the system processing delay to approximately 40 ms, corresponding to a computational efficiency improvement of 90.48%.

The proposed framework demonstrates substantial enhancements in key performance metrics, including signal fidelity, noise resistance, and real-time processing capabilities. Thus, it offers both theoretical contributions and practical applicability in weak signal detection tasks.

## Figures and Tables

**Table 1:** Experimental design for different heights.

Height	Signal-to-Noise Ratio of the Original Signal
50 cm	8 dB
100 cm	5 dB
150 cm	3 dB

**Table 2:** Comparison of time-domain metrics.

Method	Correlation Coefficient	Mean Square Error	Signal-to-Noise Ratio (dB)
FIR Filtering	0.4328	0.009996745	17.10 dB
Multi-stage Filtering	0.9854	0.01331926	24.77 dB
Optimized Wavelet Transform	0.9890	0.009579688	25.83 dB
Final Output	0.9890	0.009579688	25.83 dB

**Table 3:** Effects of different noise processing techniques.

Noise Type	Original Signal (SNR)	Processed Signal (SNR)	Signal-to-Noise Ratio Improvement
Dark Current Noise	−20 dB	2.43 dB	22.43 dB
Gain Fluctuation Noise	−20 dB	4.88 dB	24.88 dB
Photo-response Non-uniformity Noise	−20 dB	4.088 dB	24.08 dB
Quantum Efficiency Fluctuation Noise	−20 dB	4.11 dB	24.11 dB
Shot Noise	−20 dB	1.19 dB	21.19 dB
Thermal Noise	−20 dB	4.67 dB	24.67 dB

**Table 4:** Signal processing effect of different noise reduction modules.

Method	Input SNR (dB)	Output SNR (dB)	Improvement Factor (dB)
FIR Filtering	−20 dB	17.10 dB	37.10 dB
Multi-stage Filtering	17.10 dB	24.77 dB	7.67 dB
Optimized Wavelet Transform	24.77 dB	25.83 dB	1.6 dB
Final Output	25.83 dB		

**Table 5:** Results of quantitative analysis of multiple sets of experimental data.

Case	Original (dB)	Processed (dB)	Changed (dB)	Coefficient of Variation	Autocorrelation Function
Case 1	0 dB	26.82 dB	26.82 dB	1.5070	0.9812
Case 2	5 dB	28.23 dB	23.23 dB	1.5061	0.9818
Case 3	10 dB	28.05 dB	17.05 dB	1.5117	0.9817
Case 4	−5 dB	28.06 dB	33.06 dB	1.4337	0.9773
Case 5	−10 dB	28.60 dB	38.60 dB	1.4980	0.9808
Case 6	−15 dB	30.36 dB	45.36 dB	1.4950	0.9823

**Table 6:** Comparison of the performance of different methods (−20 dB SNR condition).

Method	Signal-to-Noise Ratio of Original Signal	Signal-to-Noise Ratio of Processed Signal	Signal-to-Noise Ratio Improvement (dB)	Correlation Coefficient
DWT [30]	−20 dB	−15 dB	5 dB	0.175
EMD [18]	−20 dB	2.85 dB	22.85 dB	0.815
EEMD [19]	−20 dB	−8.3768 dB	11.6232 dB	0.195
LMD [31]	−20 dB	6.53 dB	26.53 dB	0.901
VMD [20]	−20 dB	0.01 dB	20.01 dB	0.301
AMHFC [32]	−20 dB	−0.06 dB	19.94 dB	0.765
Ours	−20 dB	25 dB	45 dB	0.981

## Data Availability

Data are contained within the article.
